# Impact of the COVID-19 pandemic on self-paid vaccination intentions for children: a cross-sectional study in China

**DOI:** 10.1136/bmjopen-2023-083056

**Published:** 2024-08-09

**Authors:** Shuo Wang, Junfang Xu, Jiming Zhu

**Affiliations:** 1 Zhejiang University School of Medicine, Hangzhou, China; 2 Tsinghua University, Beijing, China

**Keywords:** COVID-19, China, cross-sectional studies, public health, vaccination

## Abstract

**Abstract:**

**Objectives:**

While it is widely accepted that COVID-19 has disrupted routine vaccination globally, the long-term impact of COVID-19 on parental vaccination intentions is uncertain. This study aims to estimate whether COVID-19 impacted parental intentions for self-paid vaccines, and provides suggestions for local vaccination policy and intervention strategies accordingly.

**Methods:**

A questionnaire-based cross-sectional survey was conducted among 2212 caregivers in Zhejiang province between 22 March and 30 June 2023. The following information was collected: sociodemographic characteristics, self-paid vaccination related intentions and behaviours, and vaccine hesitancy measured by the Vaccine Hesitancy Scale. Multiple multinomial logistic regression models were used to analyse the factors influencing the change in vaccination intentions.

**Results:**

In total, 19.32% (n=390) of respondents increased their intention to immunise their children with self-paid vaccines after the COVID-19 epidemic, 9.16% (n=185) decreased their intention, and 71.52% (n=1444) of respondents indicated that the COVID-19 epidemic did not affect their intention. The major reason for increased intentions was ‘Vaccines are effective in preventing diseases’ (83.89%) and for decreased intentions was ‘Worried about the side effects of vaccines’ (65.95%). A higher hesitancy degree (OR=2.208, p=0.0001), reduced trust in vaccines after COVID-19 (OR=16.650, p<0.0001), doctors’ recommendation of Expanded Programme on Immunization vaccines (OR=2.180, p=0.0076), and non-perfect satisfaction with vaccine information (all OR>1, all p<0.05) were considered to be drivers of decreased intention.

**Conclusion:**

Although the intentions of self-paid vaccinations were not largely influenced, nearly 30% of caregivers’ vaccination intentions changed after the COVID-19 pandemic and most of them increased their intentions. In addition, vaccination history of self-paid vaccines, vaccine information, vaccine trust and doctors’ recommendations were the active factors for self-paid vaccination. Therefore, education on the knowledge of self-paid vaccines for caregivers should be implemented to increase their vaccination intentions and decrease the threat of infectious diseases to children’s health.

**STRENGTHS AND LIMITATIONS OF THIS STUDY**

- The study conducted a cross-sectional and face-to-face survey among caregivers in Hangzhou, China.
- The multiple multinomial logistic regression model was used to analyse the influencing factors.
- A modified version of the Vaccine Hesitancy Scale was used.
- There may exist potential selection bias in the study sample.
- The study didn’t examine whether change in intention is related to actual vaccination behaviour.

## Background

 Although the COVID-19 pandemic is currently under control, it has caused unprecedented impacts including parental trust in vaccines and the timeliness of vaccination.^1^ Studies from many countries (eg, the USA, Brazil and Canada) have found that the COVID-19 epidemic has made vaccination more difficult and that parents were more hesitant to routinely vaccinate their children.^2^,^4^ However, a few studies have indicated an increased intention among individuals towards vaccination against influenza.^5 6^

Immunisation has proven to be one of the most cost-effective health investments.^7^ China proposed the Expanded Programme on Immunization (EPI) in 1978.^8^ The EPI vaccines are provided free of charge and are mandatory for school enrolment, which has a very strict oversight over any vaccine waiver. Nowadays, the EPI schedule contains a primary series of vaccines for infants, including hepatitis B, BCG, polio, measles, a combination of diphtheria, tetanus and pertussis, Japanese encephalitis, meningococcal meningitis, hepatitis A, rubella and mumps. Vaccines such as varicella, influenza, rotavirus, haemophilus influenzae type B, pneumococcal vaccines, and so on, not covered by the EPI, can be received voluntarily, but the cost has to be borne by the individuals.^9^ Therefore, caregivers’ perceptions and intentions regarding self-paid vaccines significantly influence the rate of vaccination as well as the number of vaccine injections to children.

Previous studies have shown that parental decisions regarding the administration of self-paid vaccines to their children were influenced by many factors, including availability, cost of the vaccine and perception of vaccine benefits.^10^,^12^ However, there is limited research on how the COVID-19 pandemic has impacted caregivers’ intentions to vaccinate their children with self-paid vaccines. While it is evident that COVID-19 disrupted routine vaccinations globally during the pandemic, the long-term impact of COVID-19 on vaccination and parental intentions for self-paid vaccination remain uncertain, especially in China, where the culture and COVID-19 influences differ from that of other countries. This study aims to estimate whether COVID-19 impacted parental intentions for self-paid vaccines, assess whether vaccine hesitancy and demographic factors are key contributing factors to parental intentions, and provide suggestions for local vaccination policies and intervention strategies accordingly.

## Methods

###  Consent to participate

All participants signed the informed consent form included in our questionnaires. Individual confidentiality was protected as part of the management of individual information and the processing of personal data.

### Sample recruitment and data collection

A cross-sectional, face-to-face survey was conducted among caregivers between 22 March and 30 June 2023. Initially, 3 districts/counties (ie, Gongshu, Linping and Tonglu districts) were randomly selected from a total of 13 districts (district/county/city) in Hangzhou, Zhejiang province, based on the level of economic development (ie, high, medium and low). Subsequently, one to two vaccination points (community health centres, maternity and childcare hospitals) from each sample district were selected to perform the survey.

The medical staff at the vaccination clinics assisted by our researchers conducted the data collection. All caregivers who took their children aged under 6 years to get vaccinated were invited to participate in the survey. We introduced our study to caregivers and asked them if they would like to participate. Those who agreed to participate and signed written consent informs were enrolled in our survey. All participants provided written informed consent. A total of 2212 participants were investigated. Respondents who submitted incomplete or illogical responses in the questionnaire were excluded. Ultimately, 2019 participants were incorporated in the final analysis.

### Measurements

Data were collected using a paper-based structured questionnaire in the Chinese language. The questionnaire items were developed by a collaboration of experts in related fields at Zhejiang University and Tsinghua University, encompassing public health, vaccine and epidemiology. Question items were externally peer-reviewed and tested on laypersons in a pilot study to evaluate the validity of the content and the reliability of the questions (Cronbach’s α=0.814).

The questionnaire consisted of three sections: self-paid vaccination behaviours and intentions; self-paid vaccine hesitancy; and demographics of both caregivers and children. For the self-paid vaccination behaviours and intentions, dive questions were asked (1) Whether COVID-19 impacted your intention for self-paid vaccines for children, (2) Whether COVID-19 impacted your trust on vaccines, (3) Whether they had vaccinated their children with self-paid vaccines in the past, (4) Which kind of vaccines were recommended by the the doctor, and (5) Whether the vaccine information provided by the healthcare provider meets their needs. For those who increased their intentions, the reasons for this were also asked, which included ‘Vaccines are effective in preventing diseases’, ‘Better understanding on the prevention of vaccine infectious diseases during the epidemic’, ‘Vaccination recommended or required by the school’ and others. For those who decreased their intentions, the reasons for this were also asked, which consisted of ‘Do not want to go in and out of medical institutions’, ‘The family’s income drops after the epidemic’, ‘Vaccines are not effective in preventing diseases’, ‘Worried about side effects of vaccines’, and others.

For vaccine hesitancy, a modified version of the Vaccine Hesitancy Scale (VHS), developed by WHO’s Strategic Advisory Group of Experts, was used to assess how caregivers felt about self-paid vaccines during the pandemic.^13^ VHS is a 5-point Likert Scale with 10 items. The score of 5 was assigned to strongly disagree, 4 to somewhat disagree, 3 to neutral, 2 to somewhat agree and 1 to strongly agree. VHS item scores are summed to a total VHS Score. The total score ranges in possibility from 10 to 50, and a score dichotomised at ≥30 represents high vaccine hesitancy, and ≤30 represents low vaccine hesitancy.^11^

### Statistical analysis

A descriptive method was used to describe the basic characteristics of participants and vaccination intention with frequency, percentage, mean and SD. Differences in categorical variables were assessed by the x² test or Fisher’s exact test where x² test assumptions were violated, and differences in continuous variables were assessed using t-tests. Values of p<0.05 were considered statistically significant. The factors influencing the change in vaccination intentions were analysed using multiple multinomial logistic regression models (reference group was ‘No change’). All data were analysed in SAS, V.9.4 (SAS Institute, Cary, North Carolina, USA).

### Patient and public involvement

Members of the public were involved in the design, conduct, reporting and dissemination of the study.

## Results

### Sample characteristics

The average age of the interviewed parents was 34.25±5.35 years for fathers and 32.78±4.79 years for mothers. Monthly household income was highest in the range of ¥10 000 to ¥20 000 (36.18%) followed by ¥5000 to ¥10 000 (23.89%). Among the children, 51.76% were male (n=1042). Moreover, 93.75% of the children were in good health (n=1890), 5.8% were in fair health (n=117) and 0.45% were in very poor health (n=9). In addition, 29.07% were migrant children (table 1).

**Table 1:** Characteristics of the study participants

Item		Frequency	Percentage (%)
Age of father, years			
	<20	16	0.79
	20–29	341	16.89
	30–39	1328	65.78
	40–49	316	15.65
	≥50	18	0.89
Age of mother, years			
	<20	25	1.24
	20–29	505	25.01
	30–39	1296	64.19
	40–49	191	9.46
	≥50	2	0.10
Occupation of father			
	Self-employed	669	33.20
	Peasant	74	3.67
	Employee of government enterprises and public institutions	991	49.18
	Unemployed and laid-off worker	15	0.74
	Others	266	13.20
Occupation of mother			
	Self-employed	439	21.79
	Peasant	65	3.23
	Employee of government enterprises and public institutions	1037	51.46
	Unemployed and laid-off worker	243	12.06
	Others	231	11.46
Education level of father			
	Junior high school and below	184	9.21
	Senior high school	801	40.09
	University degree	842	42.14
	Master’s degree and above	171	8.56
Education level of mother			
	Junior high school and below	178	8.85
	Senior high school	778	38.69
	University degree	913	45.40
	Master’s degree and above	141	7.01
Family monthly income (*¥1000)			
	<5	95	4.75
	5~10	478	23.89
	10~20	724	36.18
	20~30	354	17.69
	>30	350	17.49
Religion			
	Non-religious	1592	78.97
	Buddhism	290	14.38
	Christianity	84	4.17
	Islam	0	0.00
	Others	50	2.48
Infectious diseases			
	Never had	1134	56.17
	Have had	885	43.83
**The child**			
Age			
	0–3 months	276	13.67
	4–6 months	250	12.38
	7–12 months	316	15.65
	13–24 months	269	13.32
	2–4 years	424	21.00
	5–6 years	484	23.97
Number of children			
	1	1030	51.02
	2	931	46.11
	≥3	58	2.87
Sex			
	Male	1042	51.76
	Female	969	48.14
Health status			
	Good	1890	93.75
	Fair	117	5.80
	Poor	9	0.45
Household registration			
	Urban	1269	63.07
	Rural	743	36.93
Migrants			
	Yes	1420	70.93
	No	582	29.07
Medical insurance			
	None	117	5.82
	UEBMI	328	16.31
	URBMI	1440	71.61
	Commercial insurance	261	12.98
	Others	103	5.12
Experience of self-paid vaccines			
	Yes	1877	93.0
	No	142	7.0

UEBMI, Urban Employee Basic Medical Insurance; URBMI, Urban Resident Basic Medical Insurance

### Impact of the COVID-19 pandemic on the vaccination intentions of self-paid vaccines

**Figure 1:**
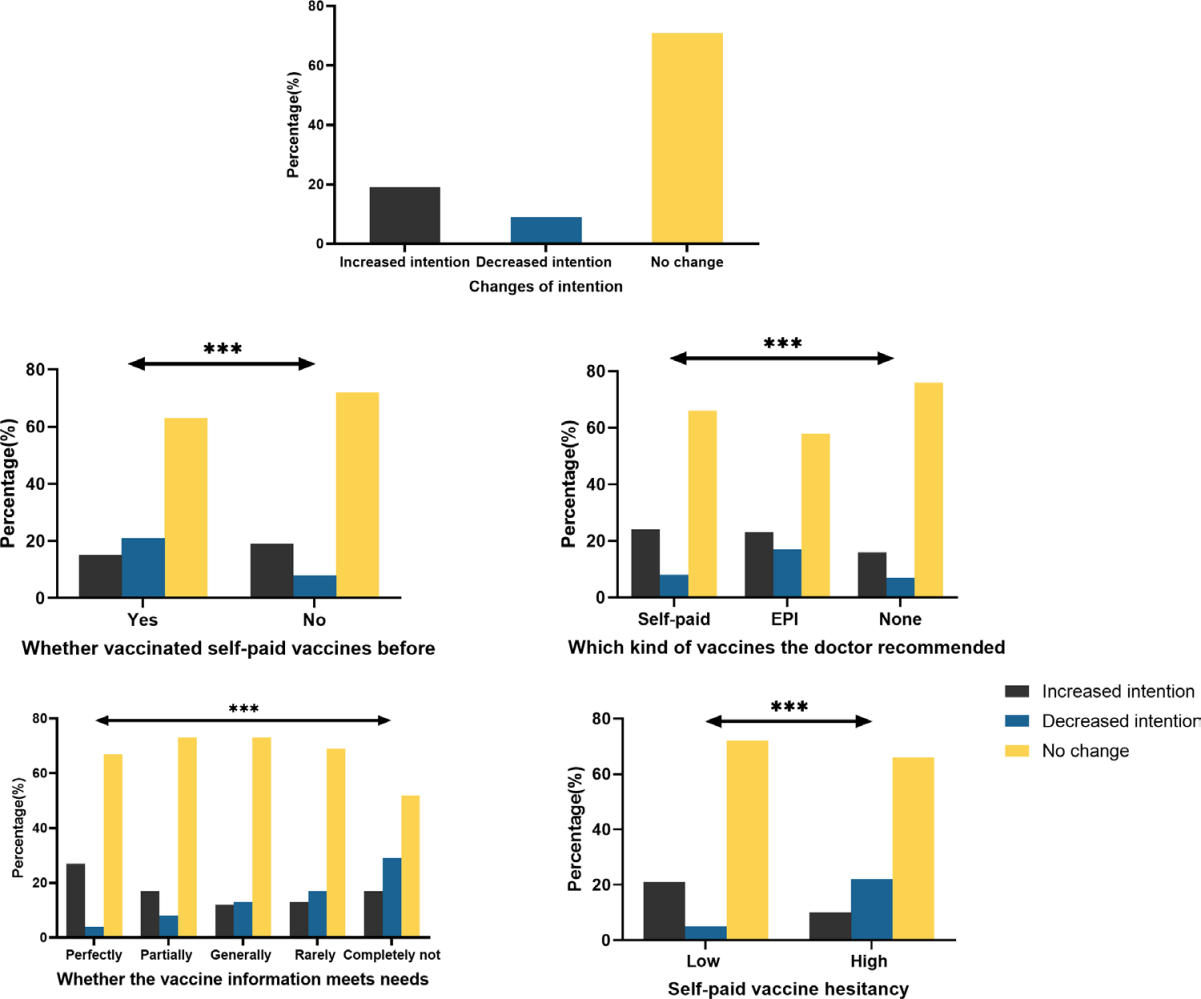
Intention changes of self-paid vaccination among participants. ***p<0.001. EPI, Expanded Programme on Immunization.

As shown in figure 1, 19.32% (n=390) of respondents increased their intention to immunise their children with self-paid vaccines, 9.16% (n=185) decreased their intention, and 71.52% (n=1444) of respondents indicated that the COVID-19 epidemic had no effect on their intention to immunise.

Respondents who had previously received self-paid vaccines were more inclined to increase their intention than those who had never vaccinated with self-paid vaccines (19.66% vs 15.22%, p<0.001), and less likely to decrease their intention than those who had not been vaccinated (8.31% vs 21.01%, p<0.001) (figure 1).

Those with a high degree of vaccine hesitancy were less likely to increase their intention to immunise their children with self-paid vaccines than those with a low level of vaccine hesitancy (10.88% vs 21.61%, p<0.001), and were more likely to decrease their intention (22.22% vs 5.61%, p<0.001) (figure 1).

### Reasons for change in intention

**Figure 2:**
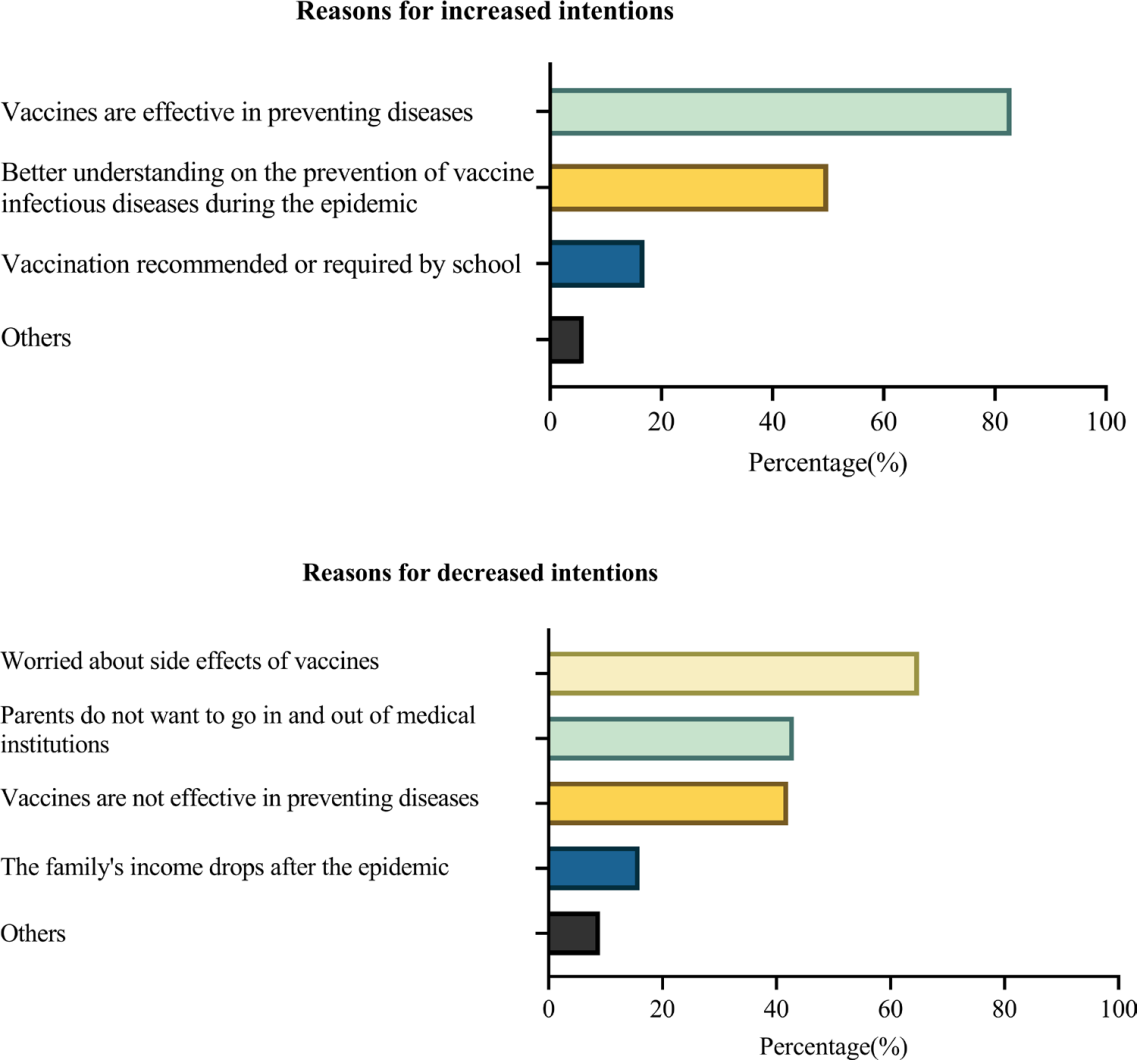
Reasons for changes in intentions for self-paid vaccines.

As shown in figure 2, the primary drivers for increased intention to receive self-paid vaccination was ‘Vaccines are effective in preventing diseases’ (83.89%), followed by ‘Better understanding on the prevention of vaccine infectious diseases during the epidemic’ (50.90%) and ‘Vaccination recommended or required by the school’ (17.44%). The main reasons for decreased intentions were ‘Worried about the side effects of vaccines’ (65.95%), followed by ‘Do not want to go in and out of medical institutions’(43.78%), ‘Vaccines are not effective in preventing diseases’ (42.70%) and ‘The family’s income drops after the epidemic’ (16.22%).

### Factors influencing changes in intentions

Table 2 shows the results of the x² test or Fisher’s exact test, which show statistical differences in the distribution of changes in vaccination intention on the age, occupations of both father and mother, religion of family members, and age of the youngest child (p<0.05). Table 3 shows the factors influencing the change in intentions regarding self-paid vaccines significantly (p<0.05). The experience of self-paid vaccines (OR=0.860, 95% CI 0.488 to 1.518), vaccine hesitancy (OR=0.678, 95% CI 0.465 to 0.989), trust on vaccines after COVID-19 (OR=8.155, 95% CI 5.992 to 11.098), doctors’ recommendation on self-paid vaccines (OR=1.540, 95% CI 1.172 to 2.023), and mother’s occupation—peasant (OR=0.340, 95% CI 0.130 to 0.888) were considered to be influencing factors of increased intentions. Conversely, respondents who had never received self-paid vaccines (OR=2.393, 95% CI 1.297 to 4.416), who had been recommended EPI vaccines (OR=2.180, 95% CI 1.230 to 3.864), those with a high vaccine hesitancy (OR=2.208, 95% CI 1.474 to 3.307), and who reduced trust in vaccines after the COVID-19 pandemic (OR=16.650, 95% CI 10.980 to 25.248) were more inclined to decrease their intention.

**Table 2:** Intentions of self-paid vaccination by different characteristics of participants

	Increased intention	Decreased intention	No change	P value
Demographic characteristics of parents				
Age of father, years				
<20	6.25	6.25	87.50	0.0411
20+	22.29	8.80	68.91	
30+	18.45	8.51	73.04	
40+	20.89	11.39	67.72	
50+	11.11	27.78	61.11	
Occupation of father				
Self-employed	21.82	10.01	68.16	0.0214
Peasant	16.22	13.51	70.27	
Employee of government enterprises and public institutions	17.96	7.57	74.47	
Unemployed and laid-off worker	6.67	26.67	66.67	
Others	19.92	10.90	69.17	
Age of mother, years				
<20	12.00	8.00	80.00	0.0471
20+	22.57	8.12	69.31	
30+	17.52	9.26	73.23	
40+	24.08	10.99	64.92	
50+	0.00	50.00	50.00	
Occupation of mother				
Self-employed	21.18	11.85	66.97	0.0299
Peasant	12.31	13.85	73.85	
Employee of government enterprises and public institutions	18.71	7.23	74.06	
Unemployed and laid-off worker	17.70	11.11	71.19	
Others	22.51	9.09	68.40	
Education level of father				
Junior high school and below	22.83	11.41	65.76	0.0905
Senior high school	19.35	10.99	69.66	
Bachelor’s degree	18.88	7.24	73.87	
Master’s degree and above	19.30	7.60	73.10	
Education level of mother				
Junior high school and below	23.60	11.80	64.61	0.4793
Senior high school	18.64	9.90	71.47	
Bachelor’s degree	18.95	8.32	72.73	
Master’s degree and above	19.86	6.38	73.76	
Family monthly income (*¥1000)				
<5	18.95	8.42	72.63	0.8107
5~10	20.29	10.67	69.04	
10~20	18.51	9.39	72.10	
20~30	18.36	7.91	73.73	
>30	20.86	7.71	71.43	
Religion				
Non-religious	19.54	8.67	71.80	0.0006
Buddhism	18.28	7.93	73.79	
Christianity	21.43	11.90	66.67	
Others	14.00	28.00	58.00	
Infectious diseases				
Never had	21.87	9.44	68.69	0.0529
Have had	16.08	8.83	75.08	
Demographic characteristics of the child				
Age				
0–3 months	21.38	6.52	72.10	0.0212
4–6 months	21.60	10.80	67.60	
7–12 months	20.25	6.01	73.73	
13–24 months	20.45	7.43	72.12	
2–4 years	16.98	8.73	74.29	
5–6 years	17.77	13.22	69.01	
Number of children				
1	18.06	8.54	73.40	0.0803
2	20.09	9.88	70.03	
≥3	31.48	5.56	62.96	
Sex				
Male	19.58	9.31	71.11	0.9151
Female	19.09	8.98	71.93	
Health status				
Good	19.68	8.73	71.59	0.0515
Fair	13.68	15.38	70.94	
Poor	22.22	22.22	55.56	
Household registration				
Urban	19.07	8.12	72.81	0.2124
Rural	19.65	11.04	69.31	
Migrants				
No	18.73	8.73	72.54	0.3166
Yes	21.31	10.31	68.38	
Medical insurance				
None	27.35	10.26	62.39	0.2737
UEBMI	18.90	8.84	72.26	
URBMI	18.96	9.24	71.81	
Commercial	19.16	9.96	70.88	
Others	11.65	11.65	76.70	

UEBMIUrban Employee Basic Medical InsuranceURBMIUrban Resident Basic Medical Insurance

**Table 3:** Factors associated with intentions to receive self-paid vaccines

Items	Increased intention				Decreased intention			
	OR	95% CI		P value	OR	95% CI		P value
		Lower	Upper			Lower	Upper	
Age of the child								
0–3 months	1.327	0.867	2.030	0.1928	0.411	0.206	0.821	0.0117
4–6 months	1.238	0.797	1.921	0.3420	0.889	0.483	1.637	0.7059
7–12 months	1.013	0.666	1.540	0.9536	0.418	0.213	0.824	0.0117
13–24 months	1.008	0.651	1.560	0.9729	0.696	0.366	1.326	0.2707
2–4 years	0.811	0.544	1.208	0.3019	0.524	0.302	0.911	0.0220
5–6 years (Ref)								
Whether vaccinated with self-paid vaccines before								
No	0.860	0.488	1.518	0.6037	2.393	1.297	4.416	0.0052
Yes (Ref)								
Trust on vaccines after COVID-19								
More trust	8.155	5.992	11.098	<0.0001	1.340	0.577	3.113	0.496
Less trust	1.782	1.157	2.747	0.0088	16.65	10.98	25.248	<0.0001
No change (Ref)								
Doctors’ recommendation or preference								
Self-paid vaccines	1.540	1.172	2.023	0.0019	1.347	0.876	2.072	0.1746
EPI vaccines	1.518	0.976	2.362	0.0639	2.180	1.230	3.864	0.0076
No recommendations (Ref)								
Satisfaction of vaccine information provided by the healthcare provider								
Perfectly satisfied (Ref)								
Partially satisfied	0.641	0.481	0.853	0.0023	2.047	1.159	3.615	0.0136
Generally satisfied	0.563	0.388	0.817	0.0025	1.966	1.068	3.620	0.0299
Rarely satisfied	0.501	0.191	1.310	0.1585	3.096	1.072	8.942	0.0368
Completely unsatisfied	0.961	0.199	4.650	0.9606	4.654	1.135	19.086	0.0327
Mother’s occupation								
Self-employed (Ref)								
Peasant	0.340	0.130	0.888	0.0276	0.539	0.192	1.515	0.2414
Employee of government enterprises and public institutions	0.893	0.647	1.231	0.4889	0.530	0.329	0.853	0.0090
Unemployed and laid-off worker	0.860	0.540	1.369	0.5249	1.288	0.676	2.453	0.4414
Others	1.311	0.836	2.057	0.2384	1.000	0.506	1.980	0.9992
Religion of caregivers								
Non-religious (Ref)								
Buddhist	0.837	0.579	1.209	0.3423	1.076	0.612	1.893	0.7988
Christianity	0.992	0.531	1.855	0.9805	1.417	0.581	3.458	0.4434
Others	0.969	0.376	2.500	0.9487	5.790	2.458	13.64	<0.0001
Vaccine hesitancy degree								
Low (Ref)								
High	0.678	0.465	0.989	0.0435	2.208	1.474	3.307	0.0001

EPIExpanded Programme on Immunization

## Discussion

This study showed that the majority of parents’ intentions to go in for self-paid vaccination was not affected by the COVID-19 pandemic (approximately 70%), and about 30% of parents changed their intention to vaccinate, of which about 20% increased and about 10% decreased. Various studies have shown divergent results: the pandemic led to increased intent to vaccinate, while for others, vaccine hesitancy had increased.^14^ Goldman *et al* showed that there was an increase of 15.8% caregivers planning to vaccinate their child against influenza the following year compared with the previous year.^15^ Another study in Saudi Arabia showed a large increase in the hesitancy rate about children’s routine vaccinations and that 45.3% of the population was identified as vaccine hesitant after the pandemic, while it was 20% in 2019 according to a previous study.^16 17^ He *et al* conducted a survey in USA and showed that caregivers’ intentions to vaccinate their children remained relatively stable (an increase of 2.7% but not statistically significant), but vaccine hesitancy has increased.^18^ One possible reason for the differences may be due to the different cultures of each country. Further investigation is needed to understand the differences between these reactions and how best to encourage self-paid vaccine acceptance.

We also found that access to vaccine information provided by doctors and doctors’ recommendations were important factors influencing parents’ intention to vaccinate. To some extent, the more information parents have about vaccines, and the stronger the doctor’s recommendation, the more willingly they receive self-paid vaccines. It suggests the substantial impact that a lack of information can have on intentions about vaccination. Likewise, a study of migrants in Europe during COVID-19 identified inadequate vaccine information, low literacy and language barriers as obstacles to routine vaccination uptake.^19^ Previous studies also showed the importance of accurate and adequate information, because misleading media reports on prevention strategies and media-related antivaccine conspiracy theories in the media during the pandemic have been shown to diminish the public willingness to vaccinate.^20^,^23^ In addition, since self-paid vaccines are optional and billed, healthcare professionals have significant influence on parental self-paid vaccine uptake, which is consistent with previous studies that healthcare providers remain the strongest influencers of vaccine decisions, both prior to and during the COVID-19 pandemic.^24^,^26^

Moreover, the previous vaccination history of self-paid vaccines was found to be a strong predictor of increased intention, aligning with findings from previous studies.^15 27 28^ Studies conducted in six countries^15^ and in England^28^ have generated comparable results, indicating that those having been vaccinated in the past against seasonal influenza are more likely to be vaccinated against pandemic influenza. It may suggest that vaccination acceptance is a habitual behaviour for individuals.^29^ One possible reason may be that, in the absence of outside intervention, a person’s self-paid vaccination behaviour arises from a deep-rooted level of cognition and consumption tendencies.

Our study revealed that 20.4% of respondents were vaccine hesitant. Previous studies have shown similar results, for example, in 2020, Kempe *et al* showed the rate of parental vaccine hesitancy to be 6.7% for routine childhood vaccines and over 25% for influenza vaccines.^4^ Santibanez *et al* reported that 20% of parents are hesitant about routine childhood vaccines.^7^ He *et al* found that routine childhood vaccine hesitancy increased during the COVID-19 pandemic.^18^ Wang *et al* found that in China parents are more hesitant about self-paid vaccines than EPI vaccines.^30^ Studies also have shown that parental vaccine hesitancy results in a decrease in vaccine uptake among children. In the European region, vaccine hesitancy has been identified as the main barrier to vaccination coverage.^31^,^33^

The occupation of the mother is the sole significant demographic factor affecting the intention to vaccinate, which suggests a correlation between mother and vaccination intention. A previous study showed the same result that the implementation of infant vaccinations is positively associated with the proportion of mothers in favour of vaccinations.^34^ This may be because the mother is mostly the child’s primary caregiver. This association suggests that immunisation providers should be mindful that vaccine perceptions vary across families with different statuses. Previous studied have shown similar results that parents with less formal education or household income may be likely to have questions or doubts about the safety of vaccines as well as the importance of all recommended childhood vaccines.^14^ For example, Boyle *et al* and He *et al* found that higher income households have higher vaccine acceptance.^18 35^ More vaccination-related education for parents less exposed to vaccine-related information, especially mothers, should be implemented to address parental unacceptance on self-paid vaccinations.

Regarding the reasons for decreased intention to have self-paid vaccines, worrying about the side effects of vaccination was the main reason. The results suggest immunisation providers need to be prepared to address concerns related to vaccine ingredients and the potential harms posed by all vaccine preventable diseases.^35^ Hence, universal health education, including the knowledge of vaccines, symptoms and probability of side effects, risk of being infected and the benefit of being immunised, should be laid significant emphasis on by healthcare institutions, governments and public media. The interplay of these factors all affect the public’s vaccine hesitancy, then affect vaccination intentions. As mentioned above, healthcare professionals hold a crucial role in influencing parental intentions of vaccine uptake. Nonetheless, many healthcare professionals reported that they felt that they were stretched with time during visits, thus they couldn’t adequately respond to caregivers’ vaccine concerns.^14^ Consequently, health education about vaccines for caregivers provided by family physicians may be a good approach. Another important way for education would be to use governmental programmes more effectively. Studies showed that vaccination was strongly associated with trust in the government.^17 36^ The community, as a grass roots government agency, plays a big role in awareness-raising and can hold lectures or talks for parents to popularise up-to-date vaccine knowledge.

### Strengths and limitations

This is the first study to investigate the impact of COVID-19 on parental intentions for self-paid vaccines in China. This study can help understand the current status of vaccination intentions that may differ from previous studies, because people may have updated their knowledge and attitudes about vaccination after the outbreak of COVID-19. Three different changes on self-paid vaccination intentions (ie, increased intention, decreased intention and no change) are considered, and the reasons and associated factors for increased intention and decreased intention are investigated. This can help deal with self-paid vaccine promotion more efficiently because targeted interventions can be provided for different people.

This study has several limitations. For example, there may exist potential selection bias in the study sample considering the survey was only conducted based on one province. Moreover, although the change of vaccination intentions of self-paid vaccines was discussed, we didn’t examine whether these changes related with actual vaccination behaviour.

### Conclusion

Although the intentions for self-paid vaccinations were not influenced largely, nearly 30% of caregivers’ vaccination intentions changed after the COVID-19 pandemic and most of them increased their intentions. In addition, vaccination history of self-paid vaccines, vaccine information, vaccine trust and doctors’ recommendations were the active factors influencing self-paid vaccination. Therefore, caregivers should be educated on self-paid vaccines to increase their vaccination intentions and decrease the threat of infectious diseases to children’s health.

## Data Availability

Data are available upon reasonable request.
