# Supplementary material for: Impact of the COVID-19 pandemic on self-paid vaccination intentions for children: a cross-sectional study in China
Source: BMJ Open. 2024 Aug 9;14(8):e083056. doi: 10.1136/bmjopen-2023-083056 (PMC11331887; doi:10.1136/bmjopen-2023-083056)
Supplement: Supplementary file 1 [file bmjopen-2023-083056.reviewer_comments.pdf]

PEER REVIEW HISTORY

BMJ Open publishes all reviews undertaken for accepted manuscripts. Reviewers are asked to complete a checklist review form (**http://bmjopen.bmj.com/site/about/resources/checklist.pdf**) and are provided with free text boxes to elaborate on their assessment. These free text comments are reproduced below.

ARTICLE DETAILS

**TITLE (PROVISIONAL)**	The Impact of the COVID-19 Pandemic on the Self-Paid Vaccination Intentions for Children: A cross-sectional study in China
**AUTHORS**	Wang, Shuo; Xu, Junfang; Zhu, Jiming

VERSION 1 – REVIEW

**REVIEWER**	James, Batholomew Chibuike<br>Sheffield Hallam University, Public Health
**REVIEW RETURNED**	09-Jan-2024

**GENERAL COMMENTS**	Methods:<br>1. Please explain what u mean by March 31 and June 2023, it not well explained, do you mean that the survey was conducted between March 31 to June 2023.<br>2. The questionnaire was designed originally for this survey or was adopted, if designed, how was it validated, please state all the process of the tool validation in methodology section. How was it pilot tested.<br>3. The ethical clearance and informed consent should be clearly stated with a subheading under the heading of methods.<br>4. The language of which the questionnaires were sampled should be stated even though it's within a community that understands a particular language.<br>5. The final number of the participant should be written in words and put 2019 in bracket to avoid confusion by the reader.<br>6. Vaccine Hesitancy Scale was adopted and modified, please clarify if permission is given to you to modify the tools. (in case of major modification) , please state it<br>7. The VHS, how did you determine the scores, you mentioned, you calculated the total score of what? If the highest score of the scale is 50, how did you determine the cut-off. Normally is it by the sum of the total mean score.<br>8. The objective of this manuscript is not clearly stated for readers. Make a clear definition of the manuscript main objective, perhaps the last paragraph in the introduction, it was stated but unclear.<br>9. Define the sample size clearly in the methodology section.

**REVIEWER**	Kuodi, Paul<br>Bar-Ilan University, Azrieli Faculty of Medicine
**REVIEW RETURNED**	17-Jan-2024

**GENERAL COMMENTS**	In the discussion on line 30, replace "self-paid vaccinate", with "self paid vaccination".<br>In the body of the manuscript, add a conclusion paragraph

**REVIEWER**	Yoda, Takeshi<br>Kawasaki Medical School, Department of Public Health
**REVIEW RETURNED**	31-Jan-2024

**GENERAL COMMENTS**	Thanks to submit the article titled as 'The impact of the COVID-19 pandemic on the self-paid vaccination intentions for children: a cross sectional study in China'.<br>The concept itself is important and necessary to research, but there are many problems for this research paper. Therefore, it is difficult to accept.<br>The most important point of this paper should be what happened to self-paid vaccination intentions in COVID-19, but the methodology description is vague on this point. The questions asked in the questionnaire could be completely different depending on whether the respondents were simply asked about their willingness to be vaccinated at their own expense or whether they were asked whether the COVID-19 had changed their willingness to be vaccinated. The authors did not provide a clear indication of how the questionnaire was written and how this important question was asked. For example, if the questioner made the questions inductive, the likelihood that respondents would choose that option would increase. It should be clear what the options were for what question statement.<br>There is one other point that I have major doubts about. You mentioned that there were 2019 respondents to the question, but did you always have one couple answer the question? In Table 1, both the father's and mother's ages add up to exactly 2019 people when the frequencies are added. Does this mean that the target population for this survey does not include singles? Why? There are no written exclusion criteria for respondents, which raises the question of whether this questionnaire survey was truly randomized. Also, many characteristics were missing in Table 1 appear in Tables 2 and 3, For example, there were no information about number of kids, experiences of self-paid vaccine in the table 1, but there were in table 2 and 3. which is difficult to understand. Normally, you should clearly show the all characteristics in table 1 if you want to show "characteristics" of the study population.<br>There are too many other questions and unknowns in this study to determine at this time whether they can be linked to the conclusions drawn within this paper, and I present the result of rejection.<br>And there are some of the important questions not clear in your paper;<br>1) What is " health status" in the tables? Please clear the definitions about "good" "fair" "poor"<br>2) in the family monthly income, RMB is an abbreviation. Please show non-abbreviated name.<br>3)There were no information about children's vaccine status and former self-paid vaccine status.<br>4) You should explain about official vaccine policy in China. Are all vaccines for children in China at self-paid? I don't know which vaccines are publicly subsidized and which vaccines are mainly self-funded, because there is no background information about vaccines.<br>5) Table 3 presents both increased and decreased intention, which one do you exactly want to argue for? You don't have to present both, and if you don't focus on one or the other based on your hypothesis, your focus will be blurred.<br>6) about figure 1, there were no information about statistics. What mean the three asterisks? You described "significantly" different

	both increased and decreased group in the 'results' section at page 9, but I think it is only chi-square test for the significant test. Chi-square test only shows the differences in all groups, not specific group's differences. So you can show the exactly significant test if you want to compare one-by-one groups.

VERSION 1 – AUTHOR RESPONSE

eviewer 1:
1. Please explain what u mean by March 31 and June 2023, it not well explained, do you mean that the survey was conducted between March 31 to June 2023.
Response: We thank the reviewer for kindly pointing out the imprecise expression of dates. We revised the sentence into "A cross-sectional and face to face survey was conducted among caregivers between March 22 and June 30, 2023."

2. The questionnaire was designed originally for this survey or was adopted, if designed, how was it validated, please state all the process of the tool validation in methodology section. How was it pilot tested.
Response: We thank the reviewer for the valuable suggestion. The question items were developed based on literature review by a collaboration of experts in related fields encompassing public health, vaccine, and epidemiology. Question items were externally peer-reviewed and tested on people in a pilot study. Then, the questionnaire was revised according to the revision comments from the expert panel and the participants in the pilot. In addition, we examined the reliability and validity of the questionnaires using Cronbach's α coefficient (α = 0.814), which showed that the questionnaire exhibited high reliability and validity. We have now carefully incorporated these in the method of the revised manuscript.

3. The ethical clearance and informed consent should be clearly stated with a subheading under the heading of methods.
Response: We are very appreciated for the reviewer's suggestion. We have now added a sub-heading "Ethical approval and consent to participate" in the methods section. The ethical clearance and informed consent are stated as follows: "All participants signed the informed consent form. Individual confidentiality was protected as part of the management of individual information and the processing of personal data. The study protocol and consent procedure were approved by the Institution Review Board of Tsinghua University, China (THU01-20230199)."

4. The language of which the questionnaires were sampled should be stated even though it's within a community that understands a particular language.
Response: Thank the reviewer very much. We have now stated the language of questionnaire in the methods section as "Data were collected using a paper-based structured questionnaire in Chinese language."

5. The final number of the participant should be written in words and put 2019 in bracket to avoid confusion by the reader.
Response: Thank the reviewer for the helpful suggestion. We have now modified the expression to avoid confusion.

6. Vaccine Hesitancy Scale was adopted and modified, please clarify if permission is given to you to modify the tools. (in case of major modification), please state it
Response: Thank the reviewer for the helpful suggestion. This Vaccine Hesitancy Scale was

generated by a WHO SAGE working group and is open source. We have now explained this in the revised manuscript.

7. The VHS, how did you determine the scores, you mentioned, you calculated the total score of what? If the highest score of the scale is 50, how did you determine the cut-off. Normally is it by the sum of the total mean score.
Response: Thank the reviewer for the helpful suggestions to further improve our study. VHS is a 5-point Likert scale with 10 items. The score of 5 was assigned to strongly disagree, 4 to somewhat disagree,3 to neutral, 2 to somewhat agree, and 1 to strongly agree. VHS item scores are summed to a total VHS score. The total score ranges from 10 to 50, and then dichotomized at 30, because this value indicated hesitancy level was the midpoint of scale scores, and this dichotomy is widely used by researchers [11]. >30 represents a high vaccine hesitancy, and <=30 represents a low vaccine hesitancy. We have now carefully incorporated these in the method of the revised manuscript.

8. The objective of this manuscript is not clearly stated for readers. Make a clear definition of the manuscript main objective, perhaps the last paragraph in the introduction, it was stated but unclear.
Response: We thank the reviewer for the helpful suggestion. The main objective of this study was to estimate whether COVID-19 impacted parental intentions for self-paid vaccines, assess whether vaccine hesitancy and demographic factors are key contributing factors to parental intentions, and provide suggestions for local vaccination policy and intervention strategies accordingly. And we have revised it in the manuscript to be more clear.

9. Define the sample size clearly in the methodology section.
Response: We thank the reviewer very much for the advice. We have now defined the sample size clearly in the methodology section as follows: “A total of 2212 participants were investigated. Respondents who submitted incomplete or illogical responses in the questionnaire were excluded. Ultimately, 2019 participants were incorporated in the final analysis.”

Reviewer 2:
1. In the discussion on line 30, replace "self-paid vaccinate", with "self paid vaccination".
Response: We thank the reviewer for kindly pointing out the typos. We have now carefully read and checked the language editing of the whole manuscript.

2. In the body of the manuscript, add a conclusion paragraph
Response: Thank the reviewer for the suggestion to further improve our study. We have now added a conclusion paragraph at the end of the manuscript as follows: “Although the intentions of self-paid vaccinations were not influenced largely, nearly 30% of caregivers’ vaccination intentions changed after the COVID-19 pandemic and most of them increased their intentions. In addition, vaccination history of self-paid vaccines, vaccine information, vaccine trust, and doctors’ recommendations were the active factors for self-paid vaccination. Therefore, education on the knowledge of self-paid vaccines for caregivers should be implemented to increase their vaccination intentions and decrease the threat of infectious diseases to children’s health.”

Reviewer 3:
1.The most important point of this paper should be what happened to self-paid vaccination intentions in COVID-19, but the methodology description is vague on this point. The questions asked in the questionnaire could be completely different depending on whether the respondents were simply asked about their willingness to be vaccinated at their own expense or whether they were asked whether the COVID-19 had changed their willingness to be vaccinated. The authors did not provide a clear indication of how the questionnaire was written and how this important question was asked. For example, if the questioner made the questions inductive, the likelihood that respondents would choose that option would increase. It should be clear what the options were for what question

statement.
Response: We thank the reviewer for the valuable suggestions to further improve our study. We understand the reviewer's concerns regarding the questionnaire. We have now incorporated the question items more clearly in the method of the revised manuscript and we have also uploaded a blank version of the questionnaire used in this study translated to English as a Supporting Information file.
Data were collected using a paper-based structured questionnaire in Chinese language. The question items were developed based on literature review by a collaboration of experts in related fields encompassing public health, vaccine, and epidemiology. Question items were externally peer-reviewed and tested on people in a pilot study. Then, the questionnaire was revised according to the revision comments from the expert panel and the participants in the pilot. In addition, we examined the reliability and validity of the questionnaires using Cronbach's α coefficient (α = 0.814), which showed that the questionnaire exhibited high reliability and validity.
The questionnaire consisted of three sections: self-paid vaccination behaviors and intentions; self-paid vaccine hesitancy, and demographics of both caregivers and the child. For the self-paid vaccination behaviors and intentions, 5 questions were asked (1) whether COVID-19 impacted your intention for self-paid vaccines for children, (2) whether COVID-19 impacted your trust on vaccines, (3) whether you vaccinated their children with self-paid vaccines in the past, (4) which kind of vaccines the doctor recommended, and (5) whether the vaccine information provided by your healthcare provider meets your needs. The changes in vaccination-related intentions were measured as "Increased intention" "Decreased intention" and "No change". For those who increased their intentions, the reasons were also asked which included "Vaccines are effective in preventing diseases", "Better understanding on the prevention of vaccine infectious diseases during the epidemic", "Vaccination recommended or required by the school" and others. For those who decreased their intentions, the reasons were also asked, which consisted of "Do not want to go in and out of medical institutions", "The family's income drops after the epidemic", "Vaccines are not effective in preventing diseases", "Worried about side effects of vaccines" and others.

2.There is one other point that I have major doubts about. You mentioned that there were 2019 respondents to the question, but did you always have one couple answer the question? In Table 1, both the father's and mother's ages add up to exactly 2019 people when the frequencies are added. Does this mean that the target population for this survey does not include singles? Why? There are no written exclusion criteria for respondents, which raises the question of whether this questionnaire survey was truly randomized.
Response: Thank the reviewer for the suggestion to further improve our study. The target population for this survey are caregivers of children under six years old, included couples and singles. We did not investigate the marital status or residency of the child's parents. We had the caregiver fill in information about both parents, so the number of parents of the child was equal. Because the proportion of single childbirths in China is extremely small, and the sample size of this survey was not very large, there were no single childbirths included in this survey. All caregivers who took their children aged under six years to get vaccinated were invited to participate in the survey. We introduced our study to caregivers and asked them if they would like to participate. Those who agreed to participate and signed written consent informs were enrolled in our survey. All participants provided written informed consent forms. The inclusion criteria were as follows: the child had to be under six years old; the participant had to be a long-term and current caregiver of the child; there must sign written consent form. Participants whose child was more than six years old, who were not the caregivers, or who did not sign written consent form were excluded.

3. Also, many characteristics were missing in Table 1 appear in Tables 2 and 3, For example, there were no information about number of kids, experiences of self-paid vaccine in the table 1, but there were in table 2 and 3. which is difficult to understand. Normally, you should clearly show the all characteristics in table 1 if you want to show "characteristics" of the study population.

Response: We thank the reviewer for the helpful suggestions to further improve our study. We have now added the missing variables “Number of kids”, “Experience of self-paid vaccines” in Table 1and revised the Figure 1 in the Results section of the revised manuscript.

4. And there are some of the important questions not clear in your paper; 1) What is " health status" in the tables? Please clear the definitions about "good" "fair" "poor"
Response: The “health status” refers to the parent's own rating of their child's health based on self-perception. In the questionnaire, respondents were asked “What do you think is the health condition of your youngest child?” with three options “good” “fair”, and “poor”.

5. 2) in the family monthly income, RMB is an abbreviation. Please show non-abbreviated name.
Response: The Ren Min Bi (RMB) is the legal tender of the mainland of the People's Republic of China. Its official ISO 4217 abbreviation is CNY (ChiNa Yuan), although the more common international abbreviation is RMB (Ren Min Bi).

6. 3) There were no information about children's vaccine status and former self-paid vaccine status.
Response: We thank the reviewer very much for the suggestion. We did not ask the children’s routine vaccination status, for the mandatory policy for EPI vaccines in China. Over the past five years, the EPI vaccines have achieved a 99% vaccination rate. The self-paid vaccine status was shown in Table 1. The relationship between the change in intention to receive self-paid vaccination and former experience of self-paid vaccine was shown in Figure 1.

7. 4) You should explain about official vaccine policy in China. Are all vaccines for children in China at self-paid? I don't know which vaccines are publicly subsidized and which vaccines are mainly self-funded, because there is no background information about vaccines.
Response: Thank the reviewer for the suggestion to further improve our study. We have now added more information about official vaccine policy in China in the section of introduction as follows:
“China proposed the Expanded Program on Immunization (EPI) in 1978. The EPI vaccines are provided free of charge and are mandatory for school enrollment, which had a very strict oversight over any vaccine waiver. Nowadays, the EPI schedule contains an infant primary vaccination series of vaccines including hepatitis B, bacillus Calmette–Guérin, polio, measles, combination diphtheria, tetanus and pertussis, Japanese encephalitis, meningococcal meningitis, hepatitis A, rubella and mumps. Vaccines not covered by the EPI can be received voluntarily, but the cost is borne by individuals, such as varicella, influenza, rotavirus, haemophilus influenzae b, pneumococcal vaccines, and so on.”

8. 5) Table 3 presents both increased and decreased intention, which one do you exactly want to argue for? You don't have to present both, and if you don't focus on one or the other based on your hypothesis, your focus will be blurred.
Response: Thank the reviewer for the advice. The aim of our study was to investigate the impact of the COVID-19 epidemic on parents' intention to vaccinate children with self-paid vaccines, which could be positive, negative, or no impact. Consequently, in Table 3, we conducted a multiple multinomial logistic regression model, using "no impact" as a control, to investigate the contributing factors of the positive and negative impact. Although the intentions of self-paid vaccinations were not influenced largely, nearly 30% of caregivers’ vaccination intentions changed after the COVID-19 pandemic. Among them, 2/3 of respondents increased their intention to immunize their children with self-paid vaccines, and 1/3 decreased their intention. Combining the influencing factors of increased intention and decreased intention, we had a detailed discussion and drew a conclusion that: vaccination history of self-paid vaccines, vaccine information, vaccine trust, and doctors’ recommendations were the active factors for self-paid vaccination.

9. 6) about figure 1, there were no information about statistics. What mean the three asterisks? You

described "significantly" different both increased and decreased group in the 'results' section at page 9, but I think it is only chi-square test for the significant test. Chi-square test only shows the differences in all groups, not specific group's differences. So you can show the exactly significant test if you want to compare one-by-one groups.
Response: Thank the reviewer for the helpful suggestion. The three asterisks *** means $P < 0.001$, and we have now added the annotation in Figure 1. We used both chi-square test to test the differences in the distribution of all groups and the method of chi-square partitioning to perform comparisons between groups.
To Editor:

We are grateful to the editor for kindly giving us the opportunity to submit a revision. All valuable comments from our reviewers have been seriously considered, and wherever possible, carefully incorporated into the revision. In addition, we have made careful editorial revisions throughout the manuscript, and the language has been checked by a native English speaker. The manuscript has been edited to meet the format requirements of the journal. All major revisions have been highlighted in yellow.
We would again like to thank the reviewer and the editor for their valuable comments and suggestions, which has helped us to further improve the manuscript. We hope that the manuscript is now acceptable for publication in the journal.

VERSION 2 – REVIEW

**REVIEWER**	James, Batholomew Chibuike<br>Sheffield Hallam University, Public Health
**REVIEW RETURNED**	17-Jun-2024

**GENERAL COMMENTS**	To the best of my knowledge the manuscript should be accepted for publication. The author has responded to all comments as advised.
